# Case of Brainular Affection from an Internal Cause

**Published:** 1804-08-01

**Authors:** W. Patterson

**Affiliations:** Londonderry


### Case of Brainular Affection from an internal Cause; *communicated by* W. Patterson, M.D. *of Londonderry.*

The 14th of December, 1803, a gentleman, aged above sixty years, was suddenly attacked with a severe pain in his forehead, accompanied with so much megrim and stomach sickness, as would have caused him to fall, had he not received support. To these symptoms were added a coldness; but from the reporter I could not learn that the coldness arose to a visible shudder. He was put to bed, and recourse was had to medical advice; according to which, blood-letting pretty largely in the arm, purging, and blistering the back, legs and head, in succession, were the measures pursued.

Four days subsequent to the seizure, I was called to visit him, and found him in bed, complaining grievously of a violent pain in the forehead, together with an irksome stricture in the eye-balls and in the surrounding teguments. The functions of the brain were impaired by a degree of stupor; but when spoken to in a round tone of voice, he could give tolerably correct answers to the questions that were put to him; yet very speedily would he relapse into the same state of stupor, attended frequently with incoherent mutterings. His pulse at the wrist was unequal, labouring, and accelerated, with a tenseness in the coat of the vessel; at the same time, the temporal arteries throbbed considerably, but were uniform in their action.

The countenance was sometimes pale, sometimes reddish, and at other times suffused with a bluish tinge; the eyes were languid, and the sense of vision seemed so much diminished, that at certain periods one would suspect that it were totally lost, had it not been found that the pupils were influenced by the light of a candle directed on them at various distances. The temperature of the skin was sometimes pretty high, more frequently below the medium warmth, and generally felt languid and flaccid.

For fluids, there was sometimes an urgent thirst; but for solids, little or no appetite remained. His stomach, indeed, continued to have a loathing, and so retrograde a disposition as to approach towards vomiting, which he himself considered to proceed from vitiated bile. His bowels were sluggish, and had not emptied themselves since the operation operation of the laxative medicine, which was a space of thirty-six hours before I saw him. He was restless, tossed much in the bed; and when he did appear to get sleep, it resembled a morbid comatose state more than a salutary repose. The organs of respiration did not appear to be particularly engaged, nor were their functions so much impaired as might be expected from the acuteness of the case. The urinary organs were equally unaffected.

From the preceding phenomena, I concluded, that there existed a determination of blood to the head, with increased tension in the arteries of the part. Under this impression, I ordered local evacuations, by means of numerous leeches to the temples, and a brisk cathartic to excite and empty the bowels, as well as to promote an equilibrium in the general circulation. The first application of the leeches procured a sensible relief; and therefore it was repeated. The cathartic was not active enough in its operation, and accordingly a stronger one, composed of calomel and aloes, was soon given, and with manifest advantage. The stupor in a short time decreased, and was succeeded by a loud talkative raving, accompanied with an unconsciousness of persons and things around him; of which inattentive state a remnant continued during several days, as particularly indicated by an illusion respecting the place where he lay. The delirious condition lasted some hours, and was followed by a profound sleep, attended with a stertor resembling that of apoplexy, but distinguishable from it by several circumstances, in particular by a softness and equable movement in the pulse.

This change, which occurred within eight days, was the harbinger of convalescence, which gradually, but slowly, took place. The principal impediments in the progress were a tardy return of appetite, and an indisposition to sleep at night, both which remained nearly a month. At present he enjoys as good a share of health as his age and constitution will admit; but his eye-sight remains impaired, and he sometimes feels a degree of pain in the region of the forehead.

Considering the symptoms and phenomena of this case, I am led to conceive, that we would be justifiable in setting it down as a decided instance of apoplexy, some leading marks of which it manifestly wanted. No doubt it might, like other diseases of the brain, have terminated in a manner resembling an apoplectic catastrophe; but, if it had any shade of the apoplectic character, it certainly was rather rather of an anomalous description. It assumed so many of the features of a species of erysipelas, which takes place in the membranes and vessels of the brain, in the evening of life, that I cannot forbear classing it as a variety of that affection. I never have seen legitimate apoplexy take into its train so many symptoms of pyrexia, and terminate by so marked a crisis. Add to this, that, in the preceding spring, he had a similar attack, attended with a more tedious head-ach, which was succeeded by a salutary inflamed eruption on the face; and that, in this country, not long before the second seizure, he had a fit of the ague, to which he was subject abroad. As to the non-appearance of cutaneous inflammation, on the latter occasion, the repeated blistering might be vicarious of that phenomenon; of which the only external spontaneous token was a glutinous discharge from the conjunctivæ and edges of the eye-lids, especially those of the left side.

*July* 7, 1804.

